# Freshwater Flux Variability Lengthens the Period of the Low‐Frequency AMOC Variability

**DOI:** 10.1029/2022GL100136

**Published:** 2022-10-25

**Authors:** Fukai Liu, Jian Lu, Young‐Oh Kwon, Claude Frankignoul, Yiyong Luo

**Affiliations:** ^1^ Frontier Science Center for Deep Ocean Multispheres and Earth System (FDOMES) and Physical Oceanography Laboratory College of Oceanic and Atmospheric Sciences Ocean University of China Qingdao China; ^2^ Laboratory for Ocean Dynamics and Climate Qingdao Pilot National Laboratory for Marine Science and Technology Qingdao China; ^3^ Atmospheric Sciences and Global Change Division Pacific Northwest National Laboratory Richland WA USA; ^4^ Physical Oceanography Department Woods Hole Oceanographic Institution Woods Hole MA USA; ^5^ UMR LOCEAN Sorbonne Université/IRD/MNHN/CNRS Paris France

**Keywords:** Atlantic Meridional Overturning Circulation, freshwater flux, internal variability, subpolar North Atlantic, salinity

## Abstract

Atlantic Meridional Overturning Circulation (AMOC) exhibits interdecadal to multidecadal variability, yet the role of surface freshwater flux (FWF) variability in this AMOC variability remains unclear. This study isolates the contribution of FWF variability in modulating AMOC through a partially coupled experiment, in which the effect of the interactive FWF is disabled. It is demonstrated that the impact of the coupled FWF variability enhances the persistence of density and deep convection anomalies in the Labrador Sea (LS), thus lengthening the period of the AMOC oscillation on multidecadal timescale and suppressing its ∼30‐year periodicity. Further lead‐lag regressions illuminate that the more persistent LS density anomalies are maintained by two mechanisms: (a) The local temperature‐salinity coupling through the evaporation and (b) a downstream propagation along the East Greenland Current of the extra salinity anomaly due to the sea ice melting changes associated with an atmosphere forcing over the southern Greenland tip.

## Key Points

- The surface freshwater flux (FWF) variability lengthens the period of the Atlantic Meridional Overturning Circulation (AMOC) variability
- The contribution from salinity in generating deep convection is enhanced with the active FWF effect
- Both local and remote effects of FWF are important in modulating AMOC low‐frequency variability

## 1 Introduction

Understanding the Atlantic Meridional Overturning Circulation (AMOC) variability is of great importance given its substantial influence on the climate of the surrounding continents. Paleoclimate proxy records (e.g., Parker et al., 2015; Zhang et al., 2019) and climate models exhibit pronounced interdecadal to multidecadal AMOC variability (e.g., Bentsen et al., 2004; Delworth et al., 1993; Garuba et al., 2018; Kwon & Frankignoul, 2012; Timmermann et al., 1998; Yan et al., 2018; Zhu & Jungclaus, 2008), which is found to be closely related to the formation of density anomalies over the deep water formation (DWF) regions in the subpolar North Atlantic (e.g., Danabasoglu et al., 2019; Escudier et al., 2013; Yeager et al., 2021).

Considerable progress has been made in AMOC research in the past, yet comprehensive understanding of the driving mechanisms of the AMOC variability remains lacking (see Buckley & Marshall, 2016 for a detailed review). Numerous studies have identified roles played by various forcings and feedbacks in AMOC variability, in which freshwater flux (FWF) associated with precipitation, evaporation, and melting can directly affect sea surface salinity (SSS) and thereby the deep convection over the DWF regions. Therefore, some studies have proposed that FWF can modulate the AMOC in a significant way. For example, hosing freshwater into the North Atlantic can immediately reduce local SSS and weaken the sinking rates of DWF, causing AMOC slowdown or even shutdown (e.g., Biló et al., 2022; Brady & Otto‐Bliesner, 2011; Kostov et al., 2019; Liu, Fedorov, et al., 2020; Stocker & Wright, 1991; Thomas & Fedorov, 2019). Liu and Fedorov (2021) suggest that anomalous freshening related to Arctic sea ice loss can propagate downstream to the subpolar North Atlantic and drive an AMOC slowdown with a multidecadal delay. In addition, FWF‐induced salinity anomalies originated from the tropics (Hu & Fedorov, 2019, 2020; Vellinga & Wu, 2004) and even the Southern Ocean (Delworth & Zeng, 2012) are found to have impacts on the DWF in the subpolar North Atlantic.

While the above studies highlight the importance of the FWF, Mignot and Frankignoul (2003), Frankignoul et al. (2009), and Zhang (2017) proposed that salinity plays merely a passive role in the subpolar gyre, and AMOC variability is more influenced by the ocean dynamics rather than the FWF at decadal to longer timescales. In addition, Eden and Willebrand (2001) and Danabasoglu et al. (2019) argued that the buoyancy forcing of the AMOC by the atmosphere is largely determined by surface heat fluxes. Even the widely acknowledged relationship between the FWF hosing and AMOC slowdown is being questioned because numerical simulations showed evidence that much of the melt water fails to reach the DWF region (Condron & Winsor, 2011, 2012; Luo et al., 2016). Therefore, despite the numerous studies of AMOC and its variability, the role of the FWF in the low‐frequency variability of AMOC remains poorly understood.

As the contributions from FWF, surface heat flux, and ocean advective processes are almost concurrent for slowly evolving AMOC variability, it is difficult to disentangle their respective roles in generating the AMOC variability. To overcome this difficulty, we purposefully design a partially coupled experiment, in which the FWF is not allowed to evolve prognostically but is prescribed as its daily climatology. Thus, the effect of FWF variability from air‐sea and ice‐sea coupling at interannual and longer timescales can be cleanly isolated from other physical processes by comparison with a fully coupled run. As shown later, the FWF variability can significantly lengthen the persistence of salinity anomalies in the Labrador Sea (LS), leading to a longer timescale of the multidecadal AMOC variability. The rest of the paper is organized as follows. Section 2 describes the model and experiment design. Section 3 presents the simulated AMOC variability and examines the role of the FWF on the internal AMOC variability. Finally, a summary and discussion of our findings are given in Section 4.

## 2 Model Experiments

The climate model used in this study is the NCAR's Community Earth System Model version 1 (CESM1; Hurrell et al., 2013), with a gx1v6 global configuration that comprises an atmosphere (Community Atmosphere Model version 5), a land (Community Land Model version 4), an ocean (Parallel Ocean Program version 2), and a sea ice (Community Ice CodE) component model. For both atmosphere and land models, the horizontal resolution is a nominal 2°, with the atmospheric component discretized on 30 uneven vertical levels. The sea ice and ocean models use an irregular horizontal grid of a nominal 1° resolution but telescoped meridionally to ∼35–50 km around Greenland and the LS. Vertically, the ocean model uses a *z*‐coordinate and has 60 uneven levels with the thickness varying from 10 m near the surface to 250 m near the bottom.

**Figure 1:**
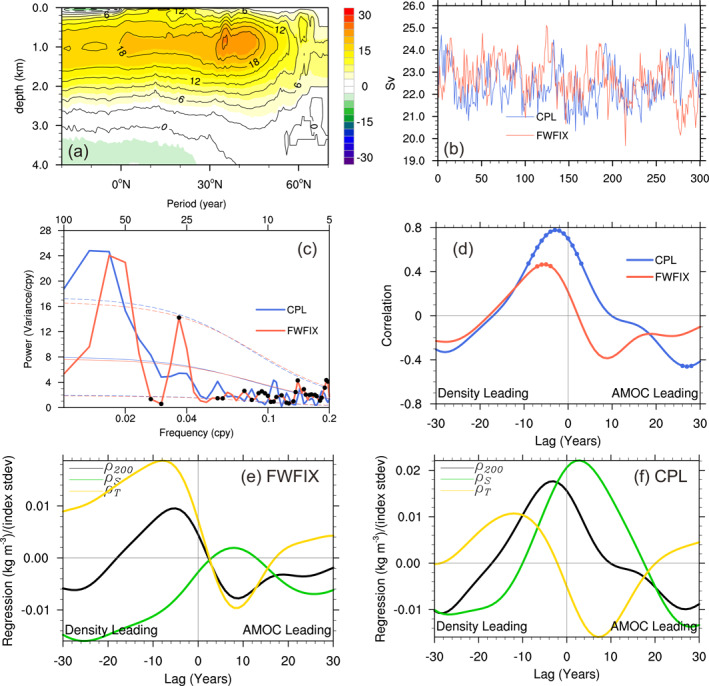
(a) Atlantic Meridional Overturning Circulation (AMOC) stream function (in Sv) averaged over the last 150 years in coupled simulation (CPL) (colors) and FWFIX (contours). Positive values indicate clockwise circulation. (b) Time series of monthly mean AMOC maximum transport at 40°N. (c) Power spectra of the AMOC PC‐1 indices from CPL (blue) and FWFIX (red). The thin solid line is a theoretical red noise spectrum obtained by fitting a first‐order autoregressive process, the dashed lines are the 95% confidence limits about the red noise spectrum. A 3‐point modified Daniell filter in the frequency domain has been applied to the spectra and the black dots indicate where the variance of the two curves is significantly different at the 90% confidence level according to Fisher's *F* test. (d) Lag correlations between upper‐ocean density ($\rho_{200}$; averaged over the top 200 m) in the Labrador Sea and the AMOC PC‐1 index with the dots indicating the lags at which the correlations are significant at 90%. (e, f) LS $\rho_{200}$ time series regressions onto the AMOC PC‐1 index in FWFIX and CPL, respectively, and the decomposition into the contribution from salinity ($\rho_{S}$) and temperature ($\rho_{T}$) (see Hassan et al., 2021 for details on the decomposition). AMOC index leads for positive lags. The time series in (b–f) are 15‐year low‐pass filtered.

The control simulation is a 1,400‐year fully coupled simulation (CPL) available from NCAR, forced with preindustrial carbon dioxide levels and solar insolation. We then conduct a partially coupled experiment (FWFIX), in which the FWF into the ocean model is substituted by the daily climatology of FWF computed from CPL simulation, while all the other coupling processes remain active. Theoretically, there are two ways the FWF variability can affect the ocean: First, FWF variability can directly affect the surface buoyancy forcing and upper ocean salinity, thereby changing vertical mixing and ocean circulation; second, the changes in ocean dynamics will in turn influence the atmosphere through air‐sea heat flux, further modulating the initial FWF forcing and influencing the ocean. In FWFIX, both these effects of FWF variability are eliminated because the ocean sees no variability in the FWF (other than the seasonal cycle). The comparison between FWFIX and the control run therefore allows us to isolate the role of the FWF variability in AMOC variability. Note that the prescribed flux components include evaporation, precipitation, runoff, and sea ice melting, thus accounting for both air‐sea and sea ice‐ocean interactions. However, the latent heat flux is not affected and evolves freely. FWFIX is initialized from the CPL equilibrium state and is integrated for 350 years, with the first 50 years being discarded as spin‐up. Note that prescribing FWF does not bring apparent climate drift: Both the mean AMOC from CPL and FWFIX show a similar maximum transport near 35°N and 1‐km depth (Figures 1a and 1b), and the deep convection strength in the central LS is almost unchanged after FWF prescribing (Figure S1b in Supporting Information S1).

This partial coupling technique has already been used to examine the formation mechanisms of the sea surface temperature (SST) response to global warming in many studies (e.g., Liu, Lu, et al., 2020; Lu & Zhao, 2012; Luo et al., 2015). Moreover, a similar approach has been employed to successfully extract the wind‐driven and buoyancy‐driven AMOC variability in CESM1 (e.g., Larson et al., 2020; Yeager & Danabasoglu, 2014), and buoyancy forcing appears to be responsible for low‐frequency variability in AMOC. In the same spirit, our experimental design will help isolate the contribution of the interactive FWF to the AMOC variability. Unless otherwise specified, all the analyses are based on annual means. The AMOC index is defined as the first principal component (PC‐1) of the overturning stream function, which has been widely used to diagnose low‐frequency variability of AMOC (e.g., Gastineau et al., 2016; Kim et al., 2018; Kwon & Frankignoul, 2012). The corresponding EOF shows a basin‐scale fluctuation of AMOC (Figure S2 in Supporting Information S1).

## 3 Results

### 3.1 Changes in AMOC Variability and Its Connection to LS Deep Convection

Figure 1c presents the power spectrum of the AMOC PC‐1 time series. As the focus of this study is on multidecadal timescales, the high‐frequency variabilities with periods less than 15 years have been filtered before the analysis. The spectral values rise above a red noise background on the multidecadal timescale in CPL, with the largest values over a broad spectral range from around 50 to 100 years. In contrast, the AMOC spectrum in FWFIX (red line) has slightly weaker and narrower band at the multidecadal timescale but an additional sharp peak at the period of ∼30 years (we refer to this peak by its periodicity as it is clearly separate from the multidecadal band). Hence, the comparison reveals that the inclusion of interactive FWF lengthens the AMOC variability on the multidecadal timescale and suppresses the ∼30‐year AMOC variability.

We next detect the connection between AMOC variability and DWF in the subpolar North Atlantic. In CESM1, both the March‐mean mixed layer depth and its standard deviation reach their maximum in the LS (Figure S1 in Supporting Information S1). By contrast, the mean depth and its variations in Nordic and Irminger Seas are much smaller, demonstrating that the LS is the primary DWF site in this model, consistent with previous CESM results (e.g., Kim et al., 2021; Kwon & Frankignoul, 2012). One concern with these results is the unrealistically strong and misplaced climatological deep convection compared to observations (Garcia‐Quintana et al., 2019), a notable mismatch between observations and climate models. Observation‐based studies usually imply a limited role of the LS in driving the climatological AMOC (e.g., Holte & Straneo, 2017; Li et al., 2021; Lozier et al., 2019; Petit et al., 2020; Pickart & Spall, 2007; Sarafanov et al., 2012; Thomas & Zhang, 2022; Zhang & Thomas, 2021). However, Oldenburg et al. (2021) found that no matter where the climatological mean deep convection occurs, the LS plays an important role in driving the low‐frequency AMOC variability. This is also true in eddy‐resolving high‐resolution models, which serve as a better analog to observations and can reproduce the observed deep convection climatology (Oldenburg et al., 2022; Yeager et al., 2021). In addition, a water mass transformation analysis following Yeager et al. (2021) also validates the driving role of LS in the low‐frequency variability of DWF and AMOC (Figure S3 in Supporting Information S1).

**Figure 2:**
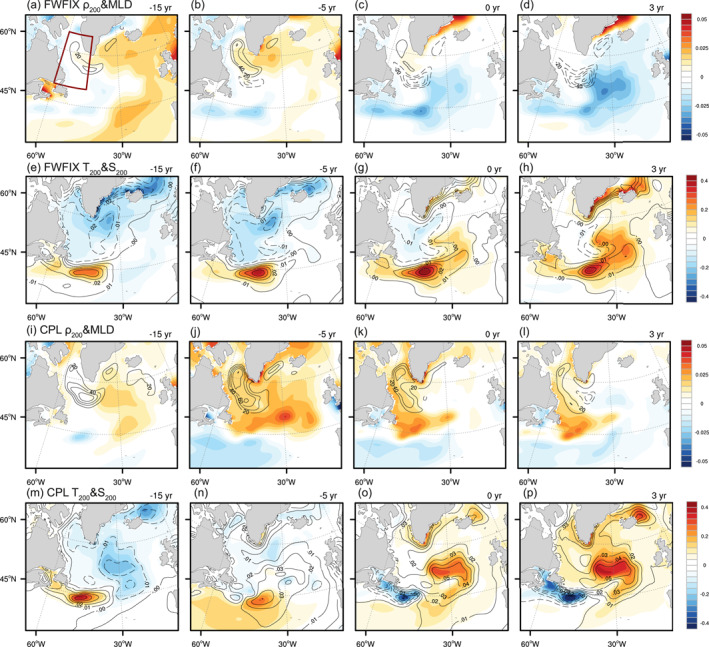
(a–d) Lag regressions of upper ocean density averaged over the top 200 m ($\rho_{200}$; $\rho_{200}$ colors; $\left(kgm^{-3}\right)/\left(index\;stddev\right)$) and March mixed layer depth (MLD, defined using a maximum buoyancy gradient criterion from Large et al. (1997); contours with CI = 20 m/(indexstddev), the zero contour is omitted) onto the Atlantic Meridional Overturning Circulation (AMOC) index for lag −15, −5, 0, and 3 years in FWFIX. (e–h) Lag regressions of temperature (colors; °C/(indexstddev)) and salinity (contours; CI = 0.01 psu/(indexstddev)) averaged over the top 200 m onto AMOC index for lag −15, −5, 0, and 3 years in FWFIX. (i–p) Same as (a–h) but in coupled simulation. AMOC index leads for positive lags. All the time series are 15‐year low‐pass filtered. The red box in (a) represents the Labrador Sea region used in this study.

Figure 1d presents the cross‐correlations between the time series of LS upper 200 m density anomalies (averaged over the red box in Figure 2a) and the AMOC PC‐1 index in both FWFIX and CPL. Note that only the anomalies in the interior LS where the ocean depth is greater than 2,000 m are considered in the following analysis. Even in the absence of the interactive FWF effect (red line), the AMOC index has an obvious lead/lag relationship with density anomalies: Dense upper ocean waters form in the LS ∼15 years prior to the AMOC peak, and the maximum correlation of ∼0.5 is obtained when density anomaly leads by 5 years, indicating the driving role of LS density anomalies. After lag zero, the positive correlation diminishes rapidly and the density anomaly switches from its maximum to its minimum within 15 years, corresponding to the pronounced 30‐year periodicity in AMOC variability (Figure 1c, red line). Figures 2a–2d presents the evolution of the upper ocean density averaged over the top 200 m related to the AMOC variations in FWFIX, which is characterized by an advective propagation along the subpolar gyre: Positive density anomalies (colors) propagate into the LS following the subpolar gyre at negative lags, leading to enhanced deep convection (contours) and AMOC spin‐up.

Are these density anomalies determined by temperature ($\rho_{T}$) or salinity ($\rho_{S}$) effect? As revealed in Figure 1e, $\rho_{T}$ and $\rho_{S}$ anomalies tend to evolve out of phase with each other, exhibiting a strong compensation between them. Overall, $\rho_{T}$ dominates the density evolution, and thus the enhanced convection is initiated by the anomalous cold water (Figures 2e and 2f). Through the damping by air‐sea heat flux, these cold temperature anomalies decay rapidly after lag −5. Meanwhile, the gradually enhanced AMOC advects warm and saline water northward along the North Atlantic Current (Figures 2f–2h, colors and contours). As the lag proceeds, the negative density anomaly moves along the cyclonic subpolar gyre, propagating downstream into the LS, reversing the phase of the 30‐year oscillation. Throughout the cycle of the low‐frequency AMOC variability in FWFIX, the density anomalies are dominated by the temperature anomalies being advected around the subpolar gyre, which are partly compensated by the opposing contribution from salinity.

With the active FWF variability in CPL, it is still the LS density anomalies that drive the AMOC variability (Figure 1d, blue line). However, the positive density anomalies generated prior to the AMOC maximum are able to persist much longer, extending even to lag 10. Compared to FWFIX (Figure 1d, red line), the duration of positive density anomalies increases by ∼50% (from ∼17 to ∼25 years), and as a result the 30‐year FWFIX oscillation almost disappears. This can also be identified in Figure 2j, where the magnitude of convection anomalies over the LS at lag −5 is nearly twice that in Figure 2b. Moreover, the positive density anomalies still occupy the interior LS 3 years after the AMOC maximum (Figure 2l), whereas in FWFIX their sign is already reversed (Figure 2d). Hence, deep convection is more persistent with active FWF effect.

Is this increased persistence still dominated by temperature contribution? According to Figure 1f, the contribution from salinity is enhanced substantially to play a more important role than temperature, particularly following the phase of maximum density: $\rho_{S}$ shows a rapid increase after lag −10 and reaches its maximum at lag 3, with twice as large an amplitude as $\rho_{T}$. Therefore, the positive density anomalies in the LS initialized by the anomalous cold temperature are extended and reinforced by salinity anomalies and persist over a much longer time, continuously driving the AMOC growth. A natural next question is what causes such persistent salinity anomalies in the LS?

### 3.2 Mechanisms of the Prolonged LS Density Anomalies Due To FWF Effect

We identify two processes through which the FWF variability can enhance and lengthen salinity anomalies in the LS: One through a local salinification associated with enhanced evaporation and the other through advection of the remote salinity anomaly produced upstream in the Irminger Sea.

#### 3.2.1 Local Mechanism

**Figure 3:**
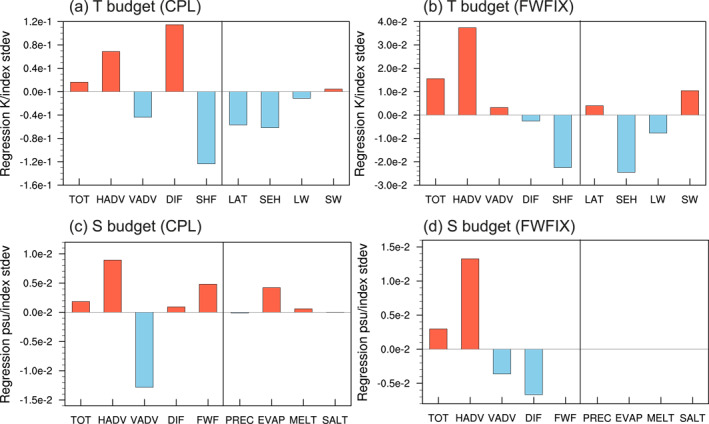
Integrated regression coefficients of the temperature budget terms onto the Atlantic Meridional Overturning Circulation PC‐1 index over lag −10 to 2 years for the Labrador Sea region in (a) coupled simulation (CPL) and (b) FWFIX. The budget terms are temperature tendency (TOT), horizontal advection (HADV), vertical advection (VADV), diffusion (DIF), surface heat fluxes (SHF) and its components due to latent heat flux (LAT), sensible heat flux (SEH), longwave radiation (LW), and shortwave radiation (SW), the units are K/(indexstddev). (c–d) Same as (a–b) but for the salinity budget terms, in which the FWF is decomposed into precipitation (PREC), evaporation (EVAP), melting flux (MELT), and salt flux due to ice formation and melt (SALT), the units are psu/(indexstddev).

To diagnose the leading mechanism for the creation and maintenance of the LS density anomalies, we first perform heat and salt budget analyses for the top 200 m in the LS following the method in Danabasoglu et al. (2012). The temperature and salinity tendency terms are maintained by the balance between surface fluxes, horizontal and vertical advection, and diffusion as a residual. Surface heat fluxes can be further decomposed into latent and sensible heat fluxes, longwave and shortwave radiations. FWF mainly comprises the precipitation, evaporation, runoff, melting flux, and salt flux due to sea ice formation/melt. For the brevity of presentation, the time series of each budget term in CPL is first regressed onto the AMOC index at different lags, and then the regression coefficients are area‐averaged over the LS and integrated from lag −10 years to lag 2 years (Figure 3). This integration interval is chosen to cover the time period when the density anomalies gain the most persistence in CPL compared to FWFIX (Figure 1d) and temperature and salinity increase monotonically in both simulations (Figures 1e and 1f). The full temporal evolutions of the regression coefficients are presented in Figure S4 in Supporting Information S1.

**Figure 4:**
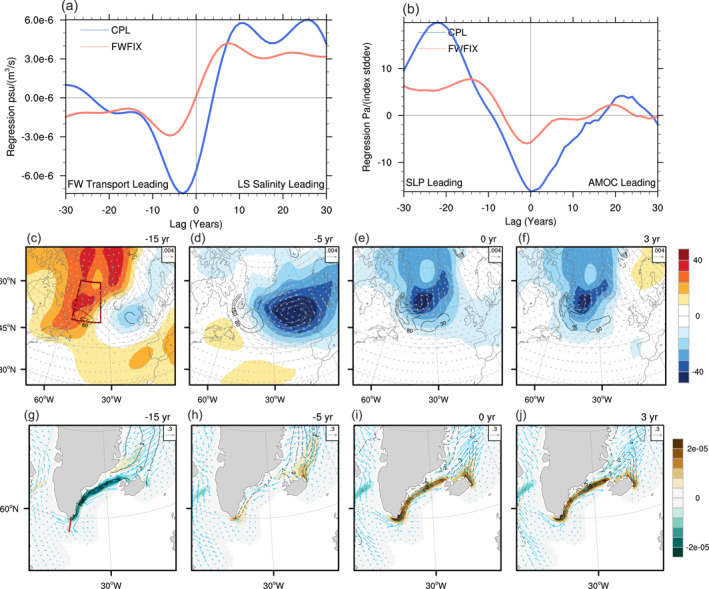
(a) Labrador Sea (LS) salinity time series regression onto the westward freshwater transport across the meridional section to the southern tip of Greenland (thick red line in (g)), the freshwater transport is calculated for the upper 300 m as $-\iint u\left(S-S_{ref}\right)/S_{ref}dxdz$, where u is the velocity perpendicular to the section, S is salinity, $S_{ref}$ is the mean salinity along the section, and positive means freshwater input into the LS. (b) Sea level pressure averaged over the southern Greenland (red box in Figure 4c) regression on the Atlantic Meridional Overturning Circulation (AMOC) index (Pa/(indexstddev)). (c–f) Regression of sea level pressure (color; Pa/(indexstddev)), upper ocean vertical diffusivity coefficient (contours with CI = 80 $\left(cm^{2}s^{-1}\right)/\left(index\;stddev\right)$; the zero contour is omitted), and surface wind stress (vector; $\left(Nm^{-2})/(index\;stddev\right)$) onto the AMOC index in coupled simulation (CPL). The vertical diffusivity coefficient is calculated using the KPP scheme in Community Earth System Model (Large et al., 1994) and is averaged over the top 200 m. (g–j) Regression of sea‐ice melting flux (color; $\left(kgm^{-2}s^{-1}\right)/\left(index\;stddev\right)$), sea ice fraction (contours; CI = 1 %/(indexstddev)), and sea‐ice velocity (vector; ($cms^{-1})/(index\;stddev)$) onto the AMOC index in CPL. Positive melting fluxes imply salinification of the surface ocean. AMOC index leads for positive lags in (c–j). All the time series are 15‐year low‐pass filtered.

In CPL, the rising temperature in the LS resulted from the positive contributions from horizontal advection and diffusion, with vertical advection and surface heat flux providing negative contributions (Figure 3a). The warm diffusion term is likely due to the intensified surface winds in the LS (Figures 4d–4f, vectors), which can stir up the upper ocean and enhance the vertical mixing between the relatively colder surface water and the warmer deeper water. This hypothesis is further supported by the changes in vertical diffusivity coefficients calculated with the KPP scheme of Large et al. (1994), which exhibit a substantial and persistent increase during lag −10 to 2 (Figures 4d–4f, contours). The important role of vertical diffusion and mixing in DWF regions in maintaining AMOC mean state and driving AMOC variability has also been identified in previous studies (e.g., Danabasoglu, 2008; Kim et al., 2021; Yang & Wen, 2020). The damping by surface heat flux suggests that, at this timescale, the ocean acts as a forcing on the atmosphere instead of the other way around. Otherwise, an active surface heat loss would have generated anomalous cooling. The two largest contributions to heat loss come from sensible heat flux and evaporative cooling. Note that the enhanced evaporation due to the rising temperature is also a component of the FWF budget, which contributes significantly to the LS salinification (Figure 3c). Therefore, despite that the rising temperature acts to decrease the upper ocean density during lag −10 to 2, the locally increased heat loss generates salinification through evaporation, which helps sustain the positive density anomalies instigated by temperature anomalies prior to lag −10.

In FWFIX, however, the relationship between evaporation and temperature anomalies is suppressed because of the prescription of the FWF with only the mean seasonal cycle and thence FWF is decorrelated with temperature, disabling the sustaining effect of the salinity‐induced density anomalies during lags −10 to 2. Therefore, both the LS temperature and salinity variations in FWFIX are controlled solely by ocean advective processes (Figures 3b and 3d), exhibiting a strong mutual cancellation (Figure 1e).

#### 3.2.2 Remote Mechanism

Apart from the local mechanism concerning surface fluxes, another major contributor to the LS salinity anomalies is the horizontal advection (Figure 3c). A natural candidate for a remote pathway for the LS salinity anomalies would be the advection of salinity anomalies from lower latitudes along the North Atlantic Current, yet it appears to be less effective in changing density than the temperature effect in both FWFIX and CPL (Section 3.1). Thus, there could exist another advective pathway through which the salinity anomalies in the LS can be sustained and reinforced even after the AMOC maximum.

A closer inspection of Figures 2m–2p suggests that the strong positive salinity anomalies (contours) around southeast Greenland starting at lag −5 might play an important role in feeding saline water to the LS and prolonging the lifespan of LS salinity anomalies. To verify this hypothesis, Figure 4a presents the LS salinity time series regressed onto the westward freshwater transport across the meridional section to the southern tip of Greenland (thick red line in Figure 4g). Clearly, the freshwater transport by the East Greenland Current into the LS is markedly reduced prior to the LS salinity maximum, with the maximum reduction of transport occurring at lag −3. This notion is further supported by the propagating signal of high salinity anomalies along the boundary of the LS (Figure S5 in Supporting Information S1), and the exchange between the boundary and the interior can be realized through interior recirculation (Biló et al., 2022; Holliday et al., 2009) and eddies (Bracco et al., 2008) that are parameterized in this model.

What is responsible for the enhanced salinity transport around the tip of Greenland, then? Figures 4c–4f present the regression of SLP (color) and wind stress anomalies (vectors) onto the AMOC index in CPL. As the lag increases, a low SLP anomaly appears in the eastern North Atlantic and moves westward toward the southern tip of Greenland. Around the AMOC maximum, the persistent negative SLP anomaly occupies the southern tip of Greenland, generating anomalous westerlies south of Greenland and anomalous southerlies over a large portion of the Irminger Sea (Figures 4e and 4f). The SLP anomaly may be driven by the SST anomalies (Figures 2o and 2p) and surface heat flux (Figure S6 in Supporting Information S1), associated with AMOC change (Frankignoul et al., 2013). As shown in Figures 4h–4j, such anomalous winds drive the sea ice northwestward toward the Denmark Strait (vectors), decrease the sea ice coverage in the Irminger Sea (contours), leaving less ice there as a potential freshwater source (colors). Such condition drives positive salinity anomalies, which are passively advected by the East Greenland Current toward the LS at 3–4 years lag (Figure 4a, blue line), boosting the magnitude of the salinification anomalies there and enhancing the AMOC timescale. Similar wind‐sea ice‐AMOC coupling has also been found in Yang et al. (2016): The weakened easterly in the Irminger Sea can result in a significant southward expansion of sea ice and thus ice melting, restraining the DWF and triggering a slowdown of the AMOC. In contrast, this positive feedback involving both the atmospheric circulation and sea ice is inactive when the FWF effect is disabled in FWFIX (Figures 4a and 4b, red lines; also see Figure S7 in Supporting Information S1).

## 4 Summary and Discussion

By prescribing the climatological FWF in a partially coupled CESM1, we examined the role of FWF variability in the low‐frequency variability of AMOC. Without the FWF variability, AMOC undergoes a pronounced 30‐year period), which is largely determined by temperature‐dominated density advection along the subpolar gyre: When colder upper ocean temperature anomalies reach the LS, they spin up the AMOC by enhancing convection strength. In turn, the strengthened AMOC transports warm temperature anomalies to the high latitudes, reversing the sign of the LS density anomalies. In this case, the salinity anomalies are almost out of phase with the AMOC‐related density variations. With the interactive FWF effect, however, the LS density anomaly is no longer dominated by temperature and the 30‐year periodicity is interrupted.

Instead, temperature and salinity in CPL both contribute to the positive density anomaly prior to and during the AMOC peak phase. In particular, the strength and duration of positive LS salinity anomaly are substantially increased, giving rise to a longer AMOC timescale. The sustained LS salinity anomaly can result from two processes:

1. The phase lag between temperature and salinity anomalies in the LS induced by the enhanced evaporation associated with the rising temperature can alter the FWF, causing surface salinification.
2. The wind‐driven northward anomalous drift of sea ice in the Irminger Sea reduces the sea ice melting there, and the resultant positive salinity anomalies are subsequently advected to the LS in 2–3 years, sustaining the LS salinity/density anomalies after the AMOC peaks.

These results demonstrate that the interdecadal (∼30 years periodicity) variability of AMOC is rooted in ocean advective processes and can be suppressed by the interactive surface FWF, while the multidecadal variability is more likely an air‐sea coupled phenomenon in which heat exchange plays an important role, as multidecadal variability exists in both CPL and FWFIX. This is in agreement with the notion of Zhu and Jungclaus (2008) that the interdecadal variability of AMOC is an ocean internal mode, while the multidecadal variability is an air‐sea coupled mode. Interestingly, through analyzing historical ocean observations, a recent study by Thomas and Zhang (2022) also proposed local and remote mechanisms that contribute to convective ventilation in the LS: A local mechanism through decadal variability of the deep convection in the LS; a remote mechanism through the vertical redistribution of remotely generated density anomalies.

We cannot rule out the possibility that some of the above results are unique to the CESM1 and may not represent the internal AMOC variability in reality, as the observational studies have suggested that the LS deep convection plays a minor role in driving the AMOC. However, the length of the observational data is too short to characterize the interdecadal to multidecadal variability of AMOC, and recent studies suggest that LS deep convection primarily regulates the low‐frequency variability of AMOC even in climate models where the climatological mean deep convection mainly occurs outside of the LS (Oldenburg et al., 2021, 2022; Yeager et al., 2021). Besides, our results point to the possibility that the observed LS density anomalies are not only generated locally but in part originate in upstream regions and are advected to the LS by ocean currents. A similar argument has been proposed by Menary et al. (2020). Another disagreement concerns whether the LS density anomalies are temperature or salinity dominated. According to Danabasoglu et al. (2019), even within a single modeling framework, the respective contributions from temperature and salinity vary across simulations. However, we used sensitivity experiments, in which we found the robust feature that the inclusion of interactive FWF will lengthen the persistency of salinity anomalies and hence density anomalies in the LS. Model resolution is another concern. The model used in this study cannot resolve eddies and the details of the circulation systems in the subpolar North Atlantic. However, Kim et al. (2021) compared a low‐resolution (nominal 1°) and a high‐resolution (nominal 0.1°) CESM1, showing that the coarse resolution model can capture the major processes responsible for the freshwater transport in the subpolar North Atlantic. Notwithstanding these deficiencies, this study is the first attempt to unveil the role of FWF variability in the low‐frequency variability of AMOC and further studies will be needed to assess the robustness of the identified mechanisms across different climate models.

## Supporting information

Supporting Information S1

## Data Availability

The postprocessed data to support the analysis are uploaded in https://doi.org/10.5281/zenodo.6690706.
