# Supplementary material for: Freshwater Flux Variability Lengthens the Period of the Low‐Frequency AMOC Variability
Source: Geophys Res Lett. 2022 Oct 25;49(20):e2022GL100136. doi: 10.1029/2022GL100136 (PMC9786630; doi:10.1029/2022GL100136)
Supplement: Supplementary file 1 — Supporting Information S1 [file GRL-49-e2022GL100136-s001.docx]

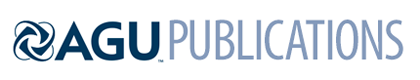


*Geophysical Research Letters*

Supporting Information for

**Freshwater flux variability lengthens the period of the low-frequency AMOC variability**

Fukai Liu^1,2*^, Jian Lu^3^, Young-Oh Kwon^4^, ﻿Claude Frankignoul^4,5^, and Yiyong Luo^1,2^

^1^ Frontier Science Center for Deep Ocean Multispheres and Earth System (FDOMES) and Physical Oceanography Laboratory, College of Oceanic and ﻿Atmospheric Sciences, Ocean University of China, 266100, Qingdao, China.

^2^ Laboratory for Ocean Dynamics and Climate, Qingdao Pilot National Laboratory for Marine Science and Technology, Qingdao, 266100, China.

^3^Atmospheric Sciences and Global Change Division, Pacific Northwest National Laboratory, Richland, WA, 99352 USA

^4^ Physical Oceanography Department, Woods Hole Oceanographic Institution, Woods Hole, MA 02543, USA

^5^ UMR LOCEAN, Sorbonne Université/IRD/MNHN/CNRS, Paris, France

Corresponding author: Fukai Liu ([fliu@ouc.edu.cn](mailto:fliu@ouc.edu.cn))

**Contents of this file**

Figures S1 to S6

**Figure S1.** (a) March-mean mixed layer depth (color; m) and its standard deviation (contours; CI is 150m) in the northern North Atlantic in CPL. The thick red line represents the location of the vertical section used in Fig. S5, and the section starts at A and ends at B. (b) Difference in March-mean mixed layer depth between FWFIX and CPL (color; m) and March-mean mixed layer depth in CPL (contours; m).

**Figure S2.** Spatial pattern of AMOC EOF1 in CPL (in Sv). Positive values indicate clockwise circulation.

**Figure S3** (a) Standard deviation of low-frequency (15-yr low-pass filtered) variability of water mass transformation (WMT) (unit: Sv) for the entire region north of 51$^{\circ}$N (TOT), Labrador Sea (LS), subpolar gyre (SPG; 51-60$^{\circ}$N, 45-5$^{\circ}$W), Irminger Sea (IMS; 60-70$^{\circ}$N, 45-5$^{\circ}$W), and Norwegian Sea (NS; 60-80$^{\circ}$N, 30-15$^{\circ}$E). Sigma2 denote ﻿density referenced to 2000 m depth; (b) Lag correlation between LS WMT in density range 36.90 to 37.10 $kg m^{-3}$ and AMOC PC-1 index, dots indicate the lags at which the correlations are significant at 90%. The time series has been 15-yr low-pass filtered. See Yeager et al. (2021) and Oldenburg et al. (2021) for details for calculating surface WMT.

**Figure S4** (a-b) Lag regressions of heat budget terms averaged over the top 200 m of the LS against the AMOC PC-1 ($K s^{-1})/(index stddev$) in CPL and FWFIX. (c-d) Same as (a-b), but for decomposition of surface heat flux. (e-f) Same as (a-b), but for salinity budget terms. (g-h) Same as (c-d), but for FWF. The shaded area marks the period (lag –10 to 2) that is used to calculate the integrated regression coefficients in Fig. 3. Note all regression coefficients in (h) are zero due to the prescribing of FWF.

**Figure S5** (a-d) Regressions of salinity onto the AMOC index along the section shown in Fig. S1a for lag -10, -5, 0 and 3 years in CPL (unit: $psu/index stddev$). (e-h) Same as (a-d), but in FWFIX. All the time series are 15-yr low-pass filtered.

**Figure S6** 15-yr low-pass filtered surface heat flux (﻿downward positive) regression onto the AMOC index for lag -15, -5, 0 and 3 years in CPL, the unit is ($W m^{-2})/(index stddev)$. AMOC index leads for positive lags.

**Figure S7** Same as Fig. 4c-f, except for FWFIX.
